# Non-dilated obstructive nephropathy

**DOI:** 10.1093/ckj/sfae249

**Published:** 2024-08-22

**Authors:** Valeria Feliciangeli, Annalisa Noce, Giulia Montalto, Stefano Germani, Roberto Miano, Anastasios D Asimakopoulos

**Affiliations:** Department of Surgical Sciences, Unit of Urology, Tor Vergata University Hospital, Rome, Italy; Department of Systems Medicine, University of Rome Tor Vergata, Rome, Italy; School of Specialization in Nephrology, University of Rome Tor Vergata, Rome, Italy; Department of Surgical Sciences, Unit of Urology, Tor Vergata University Hospital, Rome, Italy; Department of Surgical Sciences, Unit of Urology, University of Rome Tor Vergata, Rome, Italy; Department of Surgical Sciences, Unit of Urology, Tor Vergata University Hospital, Rome, Italy

**Keywords:** malignance disease, NDOU, non-dilated obstructive nephropathy, non-dilated obstructive uropathy, retroperitoneal fibrosis

## Abstract

Obstructive nephropathy (ON) is a common and reversible cause of post-renal acute kidney injury (AKI) and may be caused by a variety of conditions. It occurs when both the upper urinary tracts are obstructed, or when one tract is obstructed in patients with a solitary kidney. ON is suspected whenever there is evidence of hydronephrosis at imaging. However, not all patients with obstruction develop hydronephrosis and significant obstruction can be present in the absence of hydronephrosis. This syndrome is called non-dilated obstructive uropathy (NDOU). It accounts for about 5% of cases of urinary obstruction and the diagnosis can be challenging. The current paper provides an overview of the literature aiming to identify the main causes of NDOU and its clinical presentation, in order to clarify when to suspect it among AKI cases. A narrative review was performed due to the overall low quality of the available evidence. Only patients with post-renal AKI and a non-dilated or minimal dilation of the intrarenal collecting system were included. As evidenced by our review, NDOU is most prevalent in the fifth and sixth decades of life and affects mainly the male gender. On hospital admission serum creatinine levels are usually very high. Among the most common clinical presentations are oliguria/anuria, abdominal pain, signs of retention such as oedema or pleural effusion, and nausea/vomiting. About three out of four cases of NDOU are due to an ab-extrinsic compression of the ureters caused by retroperitoneal fibrosis or malignant disease. An effective and minimally invasive urinary diversion is obtained with ureteric stenting or a percutaneous nephrostomy. A correct diagnosis of NDOU may be challenging but it is of paramount importance as it can lead to a prompt management with a potential complete resolution of both obstruction and acute renal failure.

## INTRODUCTION

Acute kidney injury (AKI) can be attributed to pre-renal, intrinsic (renal) or post-renal causes. Pre-renal causes are usually easy to identify based on the patient's comorbidities and his/her general clinical condition. Intrinsic causes, regardless of their nature, are associated with poor clinical outcomes. Post-renal causes may include a variety of conditions having as a common denominator an impairment of the urine deflux into the bladder related to an ‘obstruction’ of the urinary system. The aetiology of the obstruction may be related to intrinsic, extrinsic or endoluminal causes (Table 1).

**Table 1:** The post-renal AKI classification.

Intrinsic causes	Extrinsic causes	DD
• Lithiasis	• Retroperitoneal and pelvic cancer	• NB
• Cancer	• Phimosis	• Drugs (SSRI)
• Clots	• Prostate hypertrophy/cancer	• iRPF
• RPN		
• Urethral stenosis		

RPN, renal papillary necrosis; DD, differential diagnosis; NB, neurological bladder; SSRI, selective serotonin reuptake inhibitors; iRPF, idiopathic retroperitoneal fibrosis.

Independent of the cause, the obstruction of a urinary system is usually suspected by the evidence of hydronephrosis or hydroureter on renal ultrasound (US) examination and/or computed tomography (CT). However, not all patients with urinary obstruction develop hydronephrosis and a significant obstruction can be present in the absence of severe hydronephrosis [1]. This is termed non-dilated obstructive uropathy (NDOU), a rare subtype of post-renal AKI, accounting for about 5% [2, 3] of cases of urinary obstruction. This syndrome can be due to different conditions such as dehydration, hypotension, severe oliguria, acute early obstruction or inability of the collecting system to dilate because of infiltrative metastatic abdominal-pelvic cancers and retroperitoneal fibrosis [4]. Approximately 60% of NDOU cases are associated with an intrapelvic malignancies (Table 2). Other less common causes include retroperitoneal fibrosis (RPF) and retroperitoneal lymphadenopathy [5].

**Table 2:** Main elements for the differential diagnosis of NDOU.

**Take-home messages**
→ Suspect NDOU in the following conditions:
- renal failure in middle-aged men
- history of abdominal-pelvic malignancy (especially metastatic cancer)
- acute onset of severe oliguria/anuria
- very high serum creatinine in presence of minimal or absent uremic symptoms

The current paper provides an overview of the literature aiming to identifying the main causes of this syndrome and its clinical presentation, to clarify when to suspect it among AKI cases. A correct diagnosis of this condition may be challenging but it is of paramount importance as it can lead to a prompt management with a potential complete resolution of both obstruction and acute renal failure.

## METHODS

A narrative review was carried out due to the overall low quality of the available evidence. Literature research was performed using PubMed, Cochrane Central and Embase databases. The research was limited to English articles published up to November 2023. Research terms included: ‘non-dilated obstructive nephropathy’, ‘non-dilated obstructive uropathy’, ‘acute kidney injury’, ‘acute kidney failure’, ‘nephropathy’ and ‘uropathy’. The reference lists of reports as well as the ‘similar articles’ feature of PubMed were also checked for additional publications.

Only patients with post-renal AKI and a non-dilated or minimal dilation of the intrarenal collecting system were included. The main parameters which were analysed and inserted in a customized database were: age, sex, symptoms and creatinine serum levels at the hospital admission, grade of hydronephrosis, aetiology of NDOU, imaging method used for diagnosis, treatment and creatinine serum levels at discharge. Only English language full-text manuscripts with exception of reviews were included. Only descriptive statistics were used.

### Study selection

**Figure 1:**
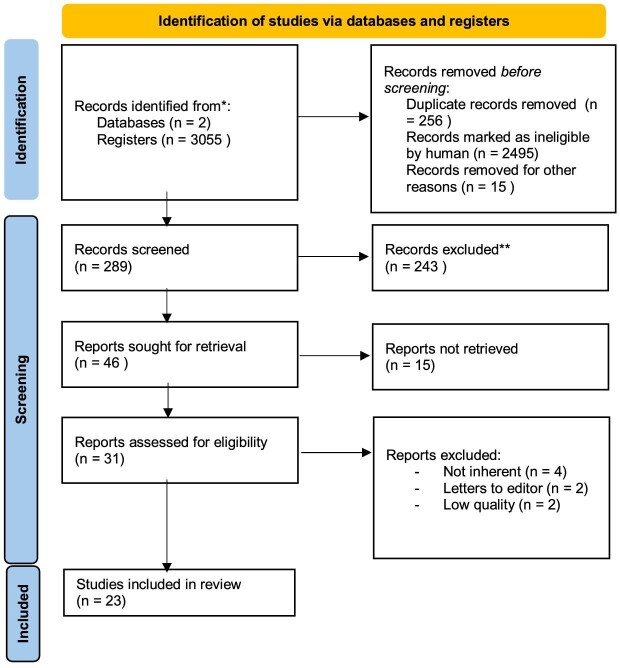
Flow of information through the different phases of this narrative review according to the PRISMA criteria.

Following an initial electronic search, 289 publications were identified through database searching as potentially eligible articles. Figure 1 provides a diagram on the flow of information through the different phases of this review according to the Preferred Reporting Items for Systematic reviews and Meta-Analyses (PRISMA) criteria. Finally, 23 manuscripts published from 1979 to 2023 that met all the inclusion and exclusion criteria were enrolled in this review. All the included papers were case reports and small cohort studies including fewer than 10 patients, for a total of 49 patients with NDOU.

## RESULTS

According to our review, NDOU is most prevalent in the fifth and sixth decades of life. Mean age at presentation is 59.3 years. It has a predominance for the male sex (80%).

Most of the patients had no dilation (73%) or minimal dilation (27%) of the calico-pyelic system when evaluated by US, CT or intravenous pyelography (IVP).

Most patients with NDOU have an extremely high creatinine level (mean value 10.5 mg/dL) on hospital admission. The most common clinical presentations are: oliguria/anuria (about 82% of patients), abdominal pain (24.5%), signs of retention like oedema or pleural effusion (16%), and vagal symptoms like nausea/vomiting (18%). Sixteen percent of patients presented with fever and 12% with haematuria. Most patients report more than one of these symptoms.

Eight percent of patients with NDOU are clinically presented with aspecific symptoms such as hypertensive crisis, palpable mass at rectal exploration, sepsis and diarrhoea. Finally, another 8% of cases of NDOU occurred in the absence of symptoms (pauci-symptomatic), with only a rise in creatinine values.

Seventy-four percent of cases of NDOU are due to an ab-extrinsic compression of the ureters caused by retroperitoneal fibrosis (8%) or metastatic malignant disease (66%) (Table 3). Among the latter, 20% of cases are secondary to metastases of prostatic cancer, 18% bladder cancer, 10% colorectal cancer, 8% lymphoma, 4% breast cancer, 4% uterine cancer and 2% pancreatic cancer (Table 3).

**Table 3:** Patients’ data.

Author and year	Patient no.	Age (years)	Sex	Sign/symptoms	Cr (mg/dl)	Grade of hydronephrosis	Aethiology
Harrison 1979 [6]	1	57	2	1	8.1	0	5
Curry 1982 [7]	2	62	2	1, 3, 4	23	1	1
	3	66	1	1, 5	15	1	2
	4	69	2	4, 6	14	1	1
Chong 1981 [8]	5	63	2	1, 7 (palpable mass at E.R.)	19.44	1	5
Rascoff 1983 [9]	6	80	2	1, 3	9.9	0	1
	7	65	2	1, 6	12.8	0	1
	8	27	2	1, 2, 4, 5, 7 (asterix, phimosis)	15.7	0	3
Carcillo 1985 [10]	9	12	1	1, 3, 5, 7 (hypertensive crisis)	12.7	0	9 (uretero-vescical stenosis)
Naidich 1986 [3]	10	71	2	1, 2	11	0	5
	11	79	2	6	8	1	2
	12	47	2	7	20	0 R,1 L	4
	13	64	2	0	9	1	4
	14	66	2	1	6	0 R, dilated L	1
	15	86	2	1	7	0	1
	16	60	2	1	8	0 R, 1 L	1
Maillet 1986 [4]	17	71	2	1	12.5	0, 0	2
	18	67	2	1, 2	11.6	0	7
	19	50	2	1, 3	14.3	0	8 (traumatic lesion of meatus)
	20	56	2	1, 2	8.8	0	7
Lyons 1988 [29]	21	73	2	1		0	2
	22	10	1	1		0	8 (post-surgical oedema)
	23	74	2	1, 6	12.6	0 R,1 L	2 (partial invasion + oedema)
Spital 1988 [11]	24	64	2	0	9.4	0	1 (oedema + necrotic debris)
	25	81	2	1, 2	11.6	0	1
	26	63	2	1, 2	13.4	1, 1	3
	27	52	2	1, 2	18.9	0	2
Spector 1989 [12]	28	30	2	1, 2, 4, 5	10.2	0, 0	5
Charasse 1991 [13]	29	48	1	1,2	10.8	0	9 (uterus)
	30	71	1	7 (septic shock + diarrhea)	7.8	0	/
	31	81	1	1, 7 (diarrhea)	4.4	1	7
	32	65	2	1, 2	10.3	0	4
	33	69	2	1, 6, 7 (penile pain)	8.9	0	1
	34	73	2	1, 2	10.8	0	3
Jarmoliński 1994 [14]	35	10	2	1, 2, 3, 4	3.92	1	7
Bhandari 1995 [15]	36	55	2	1, 2, 4, 6	11.9	0	7
Canavese 1998 [1]	37	59	2	1	1.4	0	9 (pancreatic metastatic cancer)
Leong 2003 [16]	38	31	2	1, 2	0.25	0, 0	7
Kim 2003 [17]	39	54	2	1, 2	16.5	1	7
Kulkarni 2005 [18]	40	80	1	1, 4	9.1	R dilated, 0 L	4
	41	59	2	1, 2	11.47	R dilated, 0 L	2
	42	60	2	0	6.11	R dilated, 0 L	4
Onuigbo 2009 [19]	43	56	2	1, 3, 4	9	R dilated, 0 L	2
Onuigbo 2010 [5]	44	56	2	1	9	R dilated, 0 L	2
	45	59	1	0	3.45	R chronic dilated, 0 L	9 (uterus)
	46	67	2	1, 3	4	0	9 (RAU)
Esprit 2017 [20]	47	64	2	2,3	10.5	0	3
El-Alali 2022 [21]	48	59	1	1, 2, 4	20.9	1	RPF secondary to 6
Shahzad 2022 [22]	49	50	1	1, 2	1.3	0	6
		Mean 2891/49 = 59	79% M, 21%F	1 = 40/49 (81.6%), 2 = 19/49 (38.7%), 3 = 8/49 (16%), 4 = 9/49 (18%), 5 = 8/49 (16%), 6 = 6/49 (12%), 7 = 4/49 (8%), 0 = 4/49 (8%)	521.38/49 = 10.6	0 = 41/56 (73%) 1 = 15/56 (27%)	1 = 10/49 (20%), 2 = 9/49 (18%), 3 = 4/49 (8%), 4 = 5/49 (10%), 5 = 4/49 (8%), 6 = 2/49 (4%) 7 = 7/49 (14%), 8 = 3/49 (6%) 2/49 (uterus 4%), 2% pancreas, 2% RUA, 2% ndd

Sex: 1 = female, 2 = male. Sign/symptoms: 0 = asymptomatic, 1 = anuric/oliguric, 2 = abdominal pain, 3 = oedema/retention signs, 4 = vagal symptoms (nausea), 5 = fever, 6 = haematuria, 7 = other. Grade of hydronephrosis: 0 = no hydronephrosis, 1 = slight hydronephrosis. Aetiology: 1 = prostatic cancer, 2 = bladder cancer, 3 = RPF, 4 = colorectal cancer, 5 = lymphoma, 6 = breast cancer, 7 = urolithiasis, 8 = other. L = left, R** **= right, RUA= acute urinary retention, ER= rectal examination.

Among the remaining cases, 14% of NDOU cases are secondary to urolithiasis, 8% to traumatic lesions or oedema of the ureter after endoscopic surgery, one case (2%) resulted as secondary to acute urine retention and another one (2%) was of unknown cause.

Diagnosis was made using antegrade pyelography in 53% of cases while the retrograde pyelography was used in 33% of cases. In 8% of cases, a renal biopsy was required for a definitive diagnosis.

In two case reports [9, 11] and for a total of only two patients a kidney biopsy was performed in the suspicion of an intrinsic AKI. In another case [21], the renal biopsy followed the pyelographic diagnosis of obstruction and it was performed to assess for a retroperitoneal fibrosis. It should be noted that the first two studies are published before the 1990s, when NDOU was still unacknowledged/underestimated as a clinical entity.

There were two misdiagnosed cases of NDOU (4%), the first because of the death of the patient before a definitive diagnosis and the second one [5, 6] that was solved through bladder catheterization before the obstruction had determined a calico-pyelic dilation.

Considering the absence of dilation, a placement of a JJ stent is usually considered as first-line treatment; when not feasible, a percutaneous nephrostomy is placed.

There was an important decrease in creatinine serum levels in all patients after the surgical treatment.

## DISCUSSION

NDOU was described for the first time in 1977 [22]. It is a post-renal obstructive AKI in the absence of ureteral dilation visible to diagnostic imaging (such as US, CT, IVP). Although an incidence of about 4%–5% among post-renal AKI causes [3] has been reported, some authors believe that this condition is underdiagnosed rather than uncommon [23]. The lack of high evidence studies, and the difficulty in formulating a correct and prompt diagnosis of NDOU, also due to the high false negative rate of the imaging techniques, hinder the correct estimation of the actual incidence of this clinical condition.

Several NDOU syndrome aetiologies have been reported. In most of the cases (60%) an ab-extrinsic compression of the ureters caused by RPF or malignant disease [9, 16] is identified as the cause of obstruction.

RFP [24] also known as Ormond's disease, was first described by Ormond in 1948 [36, 37]. It is an uncommon disease secondary to an inflammatory condition and deposit of fibrotic tissue that involves the retroperitoneal area over the lower four lumbar vertebrae [15, 25, 26].

Among malignancies, the most frequently associated with NDOU are metastatic lesions due to prostate cancer, bladder cancer, lymphoma, colorectal cancer and breast cancer [11, 27]. NDOU syndrome has been also described in a limited number of patients with metastatic pancreatic or uterine cancer [4, 28].

There have also been described cases of NDOU syndrome secondary to lithiasis [3, 16, 14, 17] as well as iatrogenic ones [4, 29].

As for the pathogenesis, the underlying mechanism is probably multifactorial but has not yet been fully clarified. Some authors [23] believe that the lack of dilation of the calico-pyelic system could be due to an incomplete obstruction, characterized by the fibrous infiltration of the only muscular layer in the absence of infiltration of the mucosal one and the ureteral lumen. The infiltration of the muscle would lead to a ‘functional’ obstruction determined by an alteration of the ureteral peristalsis, responsible for the lack of progression of urine into the bladder. In fact, it has been demonstrated that the peristaltic activity decreases with obstruction or infection of the ureter [30].

With acute, severe bilateral obstruction, however, there is an effect on eGFR, although it has been shown experimentally in rats that glomerular filtration does continue even in complete obstruction, but at a reduced rate because of the presence of compensatory mechanisms [31]. One of these mechanisms is the pyelosinus extravasation intended as the increasing transport through both the lymphatic and venous system of the kidney through continuous water reabsorption by the tubules so that they could decompress themselves [7, 8, 13]. This decompression would lead to the absence of dilation. This same mechanism would appear to be responsible for the failure to dilate the calico-pyelic system in case of retroperitoneal carcinomatosis [3, 32].

Further mechanisms such as mucosal oedema and cellular debris would cause an early and sudden obstruction so that renal failure would rapidly develop before dilatation could occur [1, 3, 7, 27].

Some authors [4, 9, 29] assume that the lack of dilation of the calico-pyelic system could be also due to a particular morphology of the renal pelvis. In fact, two different patterns of renal pelvis are usually described: a dendritic (ramified) pattern, which features long, slim and ramified calyces (in which the renal pelvis is often divided) and an ampullary pattern characterized by short calyces, which drain almost directly into the big and wide renal pelvis. Moreover, transition patterns between the two have been described. The ramified renal pelvis, according to these authors, would seem more frequently related to the NDOU. However, the exact pathophysiologic mechanism of this link is not adequately specified and consequently this association may be considered rather speculative.

Additional causes of NDOU syndrome may be represented by dehydration [15, 33] or a septic framework with hypotension [19].

It is interesting to report that cases of obstructive hepatopathy in the absence of dilation of the intrahepatic ducts have also been identified, and the underlying aetiology seems to be like that of NDOU syndrome [34].

As for clinical presentation, symptoms can vary according to the site, the degree and the onset of NDOU, and can be highly aspecific. As previously described, most patients present at hospital admission with symptoms that may be related to an early onset acute AKI such as oliguria and anuria, signs of retention. In addition, nausea and vomiting may also be related to acute abdominal pain if there is a complete obstruction of the urinary system, for example in cases of a ureteral stone. Moreover, a febrile status could indicate an NDOU with a supra-infection of the urinary system. Gastrointestinal symptoms, such as constipation and diarrhoea, could help to investigate a bowel obstruction or colonic mass as contributing factors to the urinary tract obstruction.

Non-contrast CT (ncCT) is the current standard to detect obstructive nephropathy. On CT, the diagnosis of obstruction is based on finding a dilatation of the collecting system above the obstruction.

Renal US is considered a viable alternative to CT for the detection of urinary tract obstruction in presence of hydronephrosis but with the advantage of being an easily available, quick examination that avoids radiation exposure of the patient.

NDOU, by definition, is a clinical syndrome related to obstructive uropathy but in the absence of dilation. For this reason, even if they are considered gold standards in the diagnosis of obstructive AKI, imaging techniques such as ncCT and US have shown insufficient sensitivity in the diagnosis of NDOU. Indeed, as reported in Table 4, when US or ncCT was performed, false negatives were recorded.

**Table 4:** Patients’ data.

Author and year	Diagnosis	Treatment	Not conclusive imaging
Harrison 1979 [6]	X	NA, death	IVP
Curry 1981 [7]	1	1	CT, US
	2	1	CT, US
	2	1	CT, US
Chong 1981 [8]	3 post-mortem	2	IVP, retrograde pyelogram
Rascoff 1983 [9]	2	1	US, renal arteriography, cystoscopy (unidentifiable ostii)
	2	1	US, IVP, CT, cystoscopy (unidentifiable ostii)
	3	1	cystoscopy, renal surgical exploration
Carcillo 1985 [10]	4	1, then 2	US
Naidich 1986 [3]	2	1	CT, US
	2	1	CT, US
	2	1, 1	US
	2	1	US
	2	1, 1	US
	2	1	US
	2	1, 1	US
Maillet 1986 [4]	2	1, 1	US, cystoscopy (unidentifiable ostii)
	2	1	Radionuclide imaging, US
	2	1, then 2	US, scintiscan (renal failure without stasis)
	2	1, then 2	US, scintiscan (renal failure without stasis)
Lyons 1988 [29]	2	1	US
	2	1	US
	2	1, 1	US
Spital 1988 [11]	2	1	CT
	3	1	US, CT, renography
	1	2, 2	US, IVP, CT
	1	2	CT, US
Spector 1989 [12]	1	1 R, 2 L	CT, US
Charasse 1991 [13]	1	2	US, X-ray
	1	2	US, X-ray
	1	2	US, X-ray
	1	2	US, X-ray
	1	2	US, X-ray
	1	2	US, X-ray
Jarmoliński 1994 [14]	2	1	US, IVP
Bhandari 1995 [15]	1	2	US
Canavese 1998 [1]	1	2	US
Leong 2003 [16]	1	2, 2	CT-99MAG3 (good perfusion, no evidence of urine formation → ATN or obstruction), US, CT
Kim 2003 [17]	1	2	US
Kulkarni 2005 [18]	2	1	CT, US
	2	1	CT, US
	2	2	CT
Onuigbo 2009 [19]	2	1	CT, US
Onuigbo 2010 [5]	2	1	US
	2	1	US
	CV Foley	CV Foley	US
Esprit 2016 [20]	1	2	CT
El-Alali 2022 [21]	3	2	CT
Shahzad 2022 [22]	1	2	CT, US
	2 = 26/49 (53%) 1 = 16/49 (33%) 3 = 4/49 (8%) 4 = 1/49 (2%) X = 1/49 (2%) CV Foley = 1/49 (2%)	1 = 35/56 (62%) 2 = 19/56 (34%) Others = 2/56 (4%)	

Diagnosis: 1 = retrograde pyelography, 2 = anterograde pyelography, 3 = biopsy, 4 = surgical exploration, X = misunderstood. Treatment: 1 = NFS (nephrostomy), 2 = stent JJ. L = left, R = right.

Harrison *et al*. 1979 [6]: this is, to the best of our knowledge, the first published manuscript on NDOU. The patient was admitted to the Emergency Department for an increase in serum creatinine levels. He was subjected to IVP that revealed a stasis of the contrast medium in the nephrogram phase in the absence of hydroureteronephrosis as for NDOU. Being a patient affected by metastatic lymphocytic lymphoma, he did not receive a drainage of the urinary tract but three sessions of whole-abdomen radiation therapy. However, the patient died and only the autopsy revealed the presence of lymphoma compressing the perivescical, perirenal and periureteral tissue. Thus, the clinical suspicion of NDOU was correct but the management was hindered by the knowledge and the means of the historical period in which the study was published. Onuigbo *et al*. 2010 [5]: this was the case of an acute urinary retention in a male subject causing NDOU that was solved through bladder catheterization; thus, no further imaging evaluations of the upper urinary tract were required.

In all cases, following the initial clinical or laboratory diagnosis of AKI, a radiologic evaluation was performed soon thereafter, and it revealed the absence of or minimal dilation. However, considering the wide range between the timing of publication of the included studies (1979–2023) that implied different clinical approaches and adoption of different imaging techniques to the same clinical condition (NDOU), it is difficult to determine whether imaging was conducted at similar stages of disease progression. Nevertheless, all these patients, regardless of the stage of disease progression, presented a severe AKI and they underwent quick surgical decompression that resolved AKI in all cases, indicating the truthfulness of the diagnosis.

The definitive diagnosis may be obtained by performing an antegrade or retrograde pyelography as reported in Table 4. Therefore, the pyelography remains nowadays the gold standard for the diagnosis of NDOU. The latter is the only imaging technique able to demonstrate the site and the presence of a urinary system obstruction.

The choice of one rather than the other diagnostic technique was usually based on the intended treatment: anterograde pyelography was chosen if the intended treatment was the percutaneous nephrostomy while retrograde pyelography was chosen if the intended treatment was the placement of a JJ stent.

Most authors [3, 4, 7, 13, 29] endorse that antegrade pyelography is more efficient than the retrograde one. The latter often requires general anaesthesia, and it could be technically difficult or impossible to perform in patients with an encasement of ureter by malignancy or in presence of ureteral stenosis. Even if more challenging, antegrade pyelography can be performed even in the absence of dilation. Complications such as bleeding or septicaemia seem rare and allow the diagnosis of NDOU and, at the same time, the immediate resolution of obstruction through the placement of a nephrostomy tube.

In 86% of the cases that were included in this review the definitive diagnosis was formulated by pyelography and in all cases, following the decompression of the obstructed renal units, the AKI was resolved, indicating a high accuracy of the diagnostic method. However, the included studies were mainly case series and consequently the sensitivity and specificity of the adopted diagnostic methods cannot be precisely evaluated.

In almost all studies, the first choice was to place an anterograde percutaneous nephrostomy, thus retrograde JJ stent, represents the second choice (62% and 34% respectively).

Concerning the criteria of choice of the method of decompression, as the reports show, these are mostly related to the impossibility of placing a retrograde JJ stent for impossibility in the visualization of the affected ureteral orifice, as well as the impossibility of placing a percutaneous nephrostomy for lack of dilation of the calico-pyelic system.

Other authors [24] claim that percutaneous nephrostomy is an invasive technique to perform in patients without a defined diagnosis of post-renal AKI. According to them, it would seem more cautious to perform a diuretic scintigraphy to prevent patients with a normal response to furosemide from undergoing a procedure that would not be of benefit. Unfortunately, literature is lacking in manuscripts concerning the use of diuretic scintigraphy among patients with obstruction and a non-dilated urinary system [35].

According to the aforementioned issues, obstructive nephropathy should be suspected in patients with no obvious cause of renal failure. In these cases, it is mandatory to perform further examinations such as retrograde/antegrade pyelography, even in cases where the imaging techniques such as US or CT do not identify hydronephrosis [1, 9].

**Figure 2:**
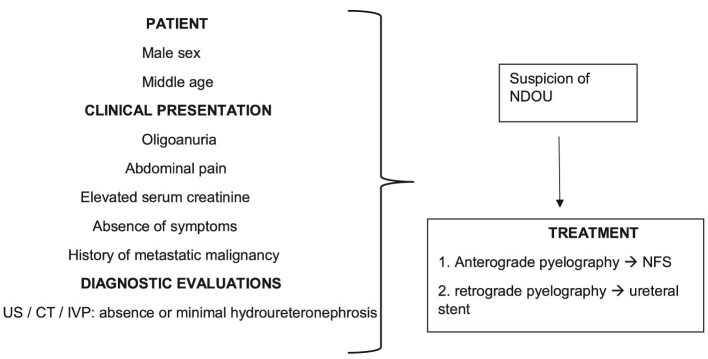
Diagnostic and therapeutic algorithm for NDOU.

Regardless of the aetiology, once a patient is diagnosed with NDOU syndrome, a drainage of calico-pyelic system with ureteral stent or nephrostomy is necessary since it allows for a rapid resolution of both obstruction and renal failure [29] (Fig. 2). In fact, in all cases analysed in this review, regardless of the choice of placing a retrograde JJ stent or percutaneous anterograde nephrostomy, except for those with post-mortem diagnosis, creatinine values dropped dramatically subsequent to the placement of a drainage.

To the best of our knowledge, this is the first review on the topic of NDOU. The main limitation concerns the quality of the included studies which are mainly observational, monocentric case reports of low quality. A great heterogeneity in the definition of ‘hydronephrosis’ and ‘absence of hydronephrosis’ is observed, hindering the inclusion of the patients in these reports. Heterogeneity is also observed in the choice of the imaging methods and of the first adopted treatment in function of the different historical period of each study. Moreover, the sample is not representative of the general population but represents the subset of hospitalized patients who received a diagnosis of AKI and the subsequent evaluation revealed NDOU. Prospective studies of patients with suspicion of NDOU are necessary to identify an accurate diagnostic algorithm for this underestimated clinical condition.

## CONCLUSION

The severity of hydronephrosis does not always correlate to the degree of obstruction. When a patient presents with an acute oliguric renal failure of unknown aetiology, the physician must vigorously pursue urinary tract obstruction and should not be dissuaded by minimal or negative findings on non-invasive imaging studies of the urinary tract. The importance of recognizing this condition is mainly due to its reversibility, with potential complete resolution of both obstruction and acute renal failure.

## Data Availability

No new data were generated or analysed in support of this research.
